# Influence of Organic Solvents on Secondary Brain Damage after Experimental Traumatic Brain Injury

**DOI:** 10.1089/neur.2020.0029

**Published:** 2020-11-06

**Authors:** Johannes Walter, Julian Schwarting, Nikolaus Plesnila, Nicole A. Terpolilli

**Affiliations:** ^1^ Institute for Stroke and Dementia Research, Munich University Hospital, Munich, Germany.; ^2^ Department of Neurosurgery, Munich University Hospital, Munich, Germany.; ^3^ Munich Cluster of Systems Neurology (SyNergy), Munich, Germany.

**Keywords:** neuroprotection, organic solvents, pre-clinical trial, traumatic brain injury

## Abstract

Many compounds tested for a possible neuroprotective effect after traumatic brain injury (TBI) are not readily soluble and therefore organic solvents need to be used as a vehicle. It is, however, unclear whether these organic solvents have intrinsic pharmacological effects on secondary brain damage and may therefore interfere with experimental results. Thus, the aim of the current study was to evaluate the effect of four widely used organic solvents, dimethylsulfoxide (DMSO), Miglyol 812 (Miglyol^®^), polyethyleneglycol 40 (PEG 40), and *N*-2-methyl-pyrrolidone (NMP) on outcome after TBI in mice. A total of 143 male C57Bl/6 mice were subjected to controlled cortical impact (CCI). Contusion volume, brain edema formation, and neurological function were assessed 24 h after TBI. Test substances or saline were injected intraperitoneally (i.p.) 10 min before CCI. DMSO, Miglyol, and PEG 40 had no effect on post-traumatic contusion volume after CCI; NMP, however, significantly reduced contusion volume and brain edema formation at different concentrations. The use of DMSO, Miglyol, and PEG 40 is unproblematic for studies investigating neuroprotective treatment strategies as they do not influence post-traumatic brain damage. NMP seems to have an intrinsic neuroprotective effect that should be considered when using this agent in pharmacological experiments; further, a putative therapeutic effect of NMP needs to be elucidated in future studies.

## Introduction

Traumatic brain injury (TBI) is a leading cause of death in children and young adults.^1,2^ Despite decades of research, therapy of secondary brain injury is still limited to a few strictly symptomatic treatment options aimed at controlling intracranial pressure (ICP) and cerebral perfusion pressure (CPP), as no interventions directly interacting with the specific pathophysiological pathways of the evolution of secondary brain damage are available.^3,4^ Evolution of brain damage following TBI is characterized by two phases: primary damage—occurring at the moment of impact—is not treatable and can only be influenced by preventive measures. In the first few hours to days after trauma a variety of pathomechanisms lead to an increase of the initial damage, termed “secondary brain damage”; the delayed nature of this process allows for therapeutic neuroprotective intervention.^5–7^ Many clinical and experimental trials evaluating a putative neuroprotective effect after trauma rely on compounds that are lipophilic and therefore need to be dispersed in solution in amphiphilic organic solvents before application *in vivo*. Results of experimental pharmacological trials in TBI vary and it has been difficult to translate promising results into clinical practice. One possible explanation might be the routine use of organic solvents, as it has yet to be systematically evaluated whether commonly used organic solvents themselves influence evolution of secondary brain damage after TBI. Therefore, the aim of the current study was to evaluate the effect of frequently used organic solvents on outcome after experimental TBI.

## Methods

All animal procedures were reviewed and approved by the Government of Upper Bavaria (protocol number 55.2-1-54-2531-132-11). The results of the study are reported according to the ARRIVE (Animal Research: Reporting of *In Vivo* Experiments) guidelines.^8^

### Animals

A total of 143 male C57 Bl/6 mice (6 to 8 weeks old, body weight 20–25 g; Charles River Laboratories, Kisslegg, Germany) were used. Animals had access to food and water *ad libitum* and were kept under a 12-h day/12-h night cycle. Post-operatively, animals were housed alone to prevent stress. Health screens and hygiene management checks were performed in accordance with Federation of European Laboratory Animal Science Associations guidelines and recommendations.^9^ As there are relevant differences between genders as far as pathophysiology is concerned and this study is intended as a proof of principle rather than a study with therapeutic intent, we performed the experiments in young male animals only to ensure comparability of the current results with previous studies using the same setup, model, and drug.

### Randomization and blinding

Animals were randomly assigned to experimental groups; test substances were assigned to experimental groups in a blinded and randomized fashion by drawing lots. Surgery, histomorphometry, and neurological testing were performed by a researcher blinded to the treatment of the animal.

### Invasive monitoring of mean arterial pressure, intracranial pressure, and cerebral blood flow

For invasive mean arterial pressure (MAP) monitoring an intra-arterial catheter was placed in the right femoral artery after open dissection of the vessel and connected to a pressure sensor (BD DTX Plus™, Becton Dickinson, Franklin Lakes, NJ, USA). Data were continuously recorded (100 Hz) and averaged every 60 sec. ICP was monitored by a parenchymal ICP sensor (Codman ICP Express, Integra Life Sciences, Ratingen, Germany) placed in the left frontal region via a burr hole. Cerebral blood flow (CBF) was measured over the middle cerebral artery territory by laser Doppler flowmetry (Periflux 5000, Perimed, Sweden) via a probe placed perpendicularly on the temporal skull. Before injection of *N*-2-methyl-pyrrolidone (NMP), CBF baseline values were recorded for 15 min; data are expressed in percent of baseline value.

### Controlled cortical impact

Experimental TBI was induced as previously described.^10–13^ In short, after right parietal craniotomy trauma was induced using a custom-made pneumatic device (velocity 8 m/sec, tip penetration depth 1 mm, contact time 150 msec). After controlled cortical impact (CCI) induction, craniotomy was closed using histoacrylic glue. To avoid hypothermia, surgery was performed on a feedback-controlled heating pad and after CCI animals were placed in an incubator at 34°C for 1 h.

### Solvents and experimental groups

**FIG. 1.**
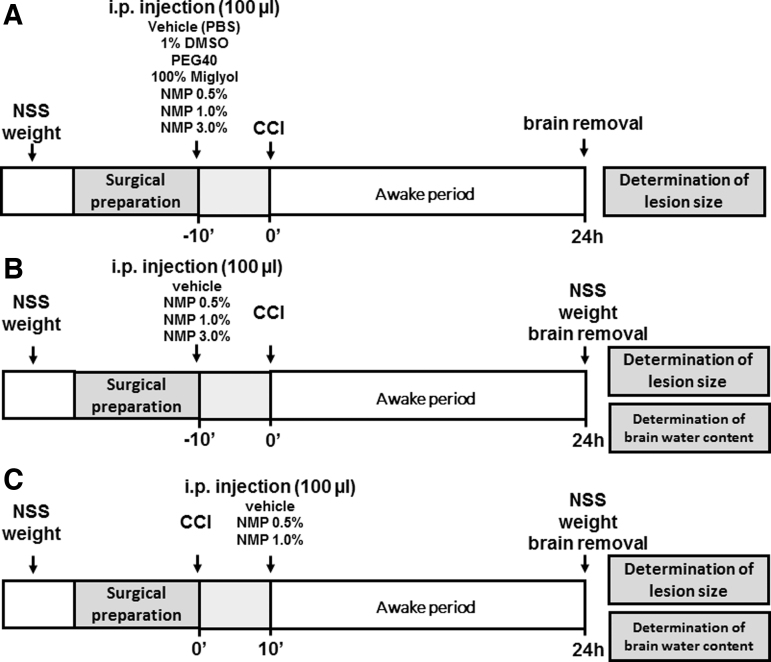
Experimental groups. **(A)** Effect of different solvents on lesion size. **(B)** Pre- and **(C)** post-traumatic application of NMP. CCI, controlled cortical impact; DMSO, dimethylsulfoxide; i.p., intraperitoneal; NMP, *N*-2-methyl-pyrrolidone; NSS, Neurological Severity Score; PBS, phosphate buffered saline; PEG 40, polyethyleneglycol 40.

In a first series, lesion volume was determined 24 h after CCI after intraperitoneal (i.p.) application of 100 μL dimethylsufoxide (DMSO) 1%, Miglyol 812 (Miglyol^®^) 100%, polyethyleneglycol 40 (PEG 40) 100%, and NMP at a concentration of 0.25%, 0.5%, 1%, or 3%, or phosphate buffered saline (PBS) as vehicle 10 min before trauma (see graphic depiction in Fig. 1A, *n* = 8 each). To further characterize NMP effects, NMP was injected 10 min before (Fig. 1B), 10 min (Fig. 1C), 1 h or 3 h after trauma induction. Control groups (15 min, 24 h) received an i.p. injection of 100 μL of PBS. Body weight was assessed in all animals in all groups 1 h before CCI and immediately before animal sacrifice (24 h after TBI). Neurological testing was done starting on the day before CCI as well as 23.5 h after CCI. Determination of brain water content (bwc) requires the whole brain, therefore brain edema was assessed in separate experimental groups.

For evaluation of the effect of NMP application on physiological parameters, 100 μL of NMP was injected at increasing concentration (0.5%, 1%, 3%, 5%, and 10%) every 15 min under continuous monitoring of MAP, ICP, and CBF after recording a 15-min baseline period. At the end of the observation period, blood was sampled from the arterial catheter for blood gas analysis (Siemens Rapidlab 348, Siemens, Munich, Germany).

### Histomorphometry

Brains were removed and 14 10-μm thick coronal sections were prepared every 500 μm starting 1000 μm behind the olfactory bulb using a cryostat (Cryostar MH 560, Microm, Walldorf, Germany). Sections were stained according to Nissl and photographed with a digital camera system at 12.5-fold magnification. Contusion volume was determined using an image analysis software as previously described^10,13^ according to the following formula:

$$V_{n}=A_{1}\times 0.5+A_{2}\times 0.5\ldots +A_{14}\times 0.5$$

### Brain water content

bwc was determined as previously described using the wet-dry method.^13^ After cervical dislocation brains were removed and wet weight was determined for each hemisphere. After storing the samples at 100°C for 24 h, dry weight was determined. The bwc was calculated using the following formula:

$$bwc\;\left(\%\right)=\frac{\left(wet\;weight\;\left(g\right)-dry\;weight\;\left(g\right)\right)}{wet\;weight\;\left(g\right)\times 100}$$

Increase in bwc was then calculated using the following formula:

$$Increase\;in\;bwc\;\left(\%\right)=bwc_{postop}\left(\%\right)-bwc_{preop}\left(\%\right)$$

and normalized to the non-traumatized hemisphere.

### Outcome

Animals were weighed pre-operatively and 24 h after trauma induction using a precision scale (Mettler-Toledo GmbH, Giessen, Germany). Neurological function was evaluated using the Neurological Severity Score (NSS), which evaluates motor function, orientation, awareness, reflexes, coordination, and gait.^10,11^ Scores range from 0 to 20 with higher scores indicating more severe neurological impairment. NSS was determined 4–6 h prior to surgery and 24 h after TBI. Animals scoring 2 or more points pre-operatively were excluded from randomization. Results are presented as the difference between pre-trauma and post-trauma scores.

### Statistical analysis

All calculations were performed using a standard statistical software package (SigmaStat 12.0, Jandel Scientific, Erkrath, Germany). Sample size was calculated based on the following parameters: alpha error = 0.05, beta error = 0.2, standard deviation (SD) of 25–30% of the mean, a minimally detectable difference of means in the primary outcome parameter (secondary lesion growth) of at least 50%, and a power of 0.8. The determined minimum group size was six to eight animals per group. For study 1 (Fig. 1A), the group number of the 15-min and 24-h control groups was increased to 14 to guarantee adequate randomization as experiments had to be performed in several blocks. For weight loss and neuroscore assessment in the pre-treatment group (Fig 1B), data from the brain edema and the lesion volume groups were pooled for control, the NMP 0.5%, and the NMP 1% group.

Due to small sample size, we used non-parametrical tests even when data passed normality testing (Kolmogorov-Smirnov method) to not overestimate a possible effect. For multiple group comparison, analysis of variance (ANOVA; Kruskal-Wallis analysis) was performed, Dunn's method was used as a post hoc test for multiple comparison versus the control (PBS, 24 h) group. Data are presented as mean ± SD as well as individual values. A statistically significant difference was assumed at *p* ≤ 0.05.

## Results

**FIG. 2.**
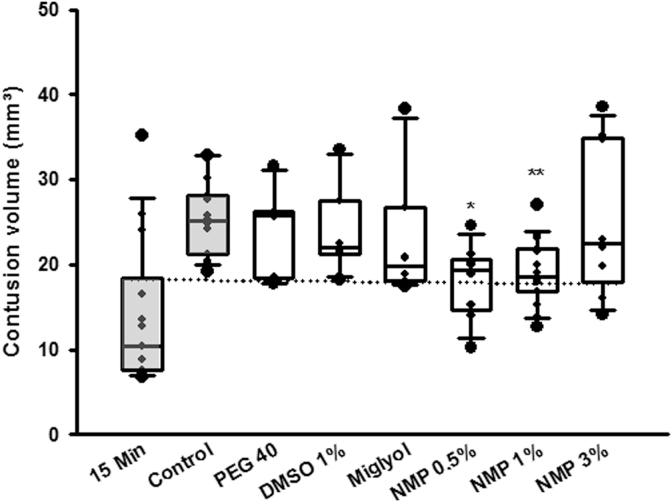
Lesion volume 24 h after CCI after injection of different organic solvents. Mean ± SD, *n* = 14 for 15-min and control groups; *n* = 8 all other groups. *p = 0.01 versus control, **p < 0.001 versus control. CCI, controlled cortical impact; DMSO, dimethylsulfoxide; Miglyol, Miglyol 812; NMP, *N*-2-methyl-pyrrolidone; PEG 40, polyethyleneglycol 40; SD, standard deviation.

**FIG. 3.**
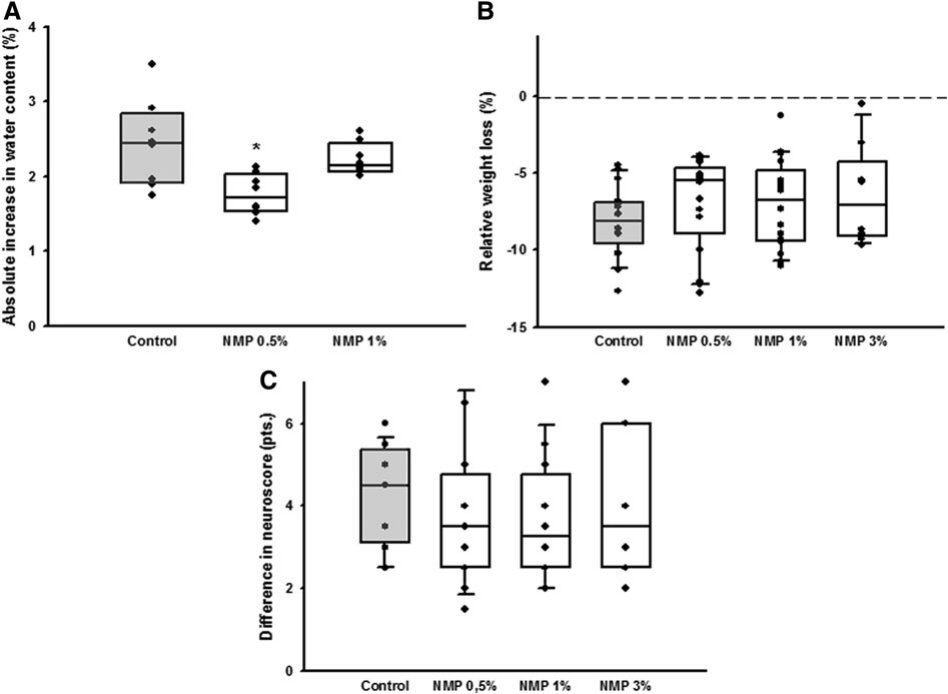
Effect of NMP on secondary brain damage—pre-treatment. **(A)** Increase in brain water content 24 h after trauma is significantly reduced in animals receiving 0.5% NMP; higher doses lead to a reduction by trend only. Mean ± SD, *n* = 8 each, *p = 0.01 versus control. **(B)** Weight loss and **(C)** performance in the Neurological Severity Score are not significantly altered by NMP application (mean ± SD, *n* = 16 for control, NMP 0.5, and NMP 1%, *n* = 8 for NMP 3%). NMP, *N*-2-methyl-pyrrolidone; SD, standard deviation.

**FIG. 4.**
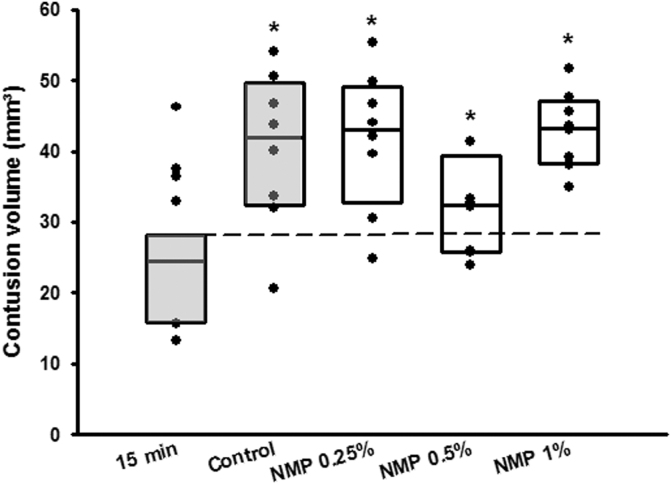
Post-treatment with NMP (applied 1 h after CCI) did not have an effect on lesion volume. Mean ± SD, *n* = 6 for 15-min group; *n* = 8 all other groups. CCI, controlled cortical impact; NMP, *N*-2-methyl-pyrrolidone; SD, standard deviation.

**FIG. 5.**
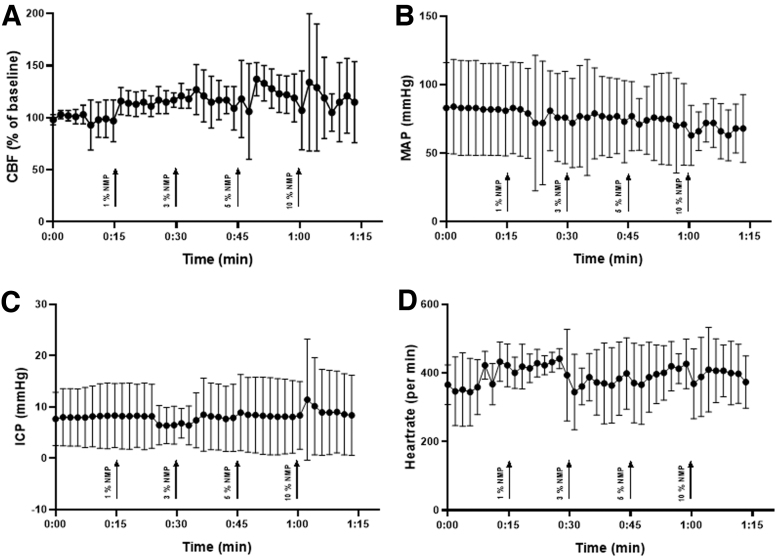
NMP injected intraperitoneally at different concentrations did not have an effect on CBF **(A)**, MAP **(B)**, ICP **(C)**, or heart rate **(D)** (mean ± SD, *n* = 5). CBF, cerebral blood flow; ICP, intracranial pressure; MAP, mean arterial pressure; NMP, *N*-2-methyl-pyrrolidone.

All animals survived to the end of the observation period; no animal was excluded from analysis. Contusion volume 15 min after CCI was 15.0 ± 2.4 mm^3^ and significantly expanded to 25.5 ± 1.2 mm^3^ 24 h after trauma induction in the vehicle (PBS) control group; (*p* = 0.003, Fig. 2); secondary lesion expansion therefore amounted to 10.5 mm^3^ or 41.2% of contusion volume 24 h after CCI. Lesion volume 24 h after CCI increased significantly in the DMSO, PEG 40, and Miglyol groups compared with the 15-min control (*p* = 0.05; *p* = 0.02; *p* = 0.04 respectively, Fig. 2) but did not differ from the control/vehicle group (24.1 ± 2.3 mm^3^; 24.3 ± 2.1 mm^3^; 23.4 ± 3.3 mm^3^, respectively (Fig. 2). Unexpectedly, application of NMP at a concentration of 0.5 and 1% significantly reduced contusion volume compared with control animals (0.5%: 18.0 ± 1.6 mm^3^, *p* = 0.001; 1.0%: 19.2 ± 1.1 mm^3^, *p* < 0.001; Fig. 2). A further increase of the NMP dose (3%) did not have an effect on lesion size. To further analyze this neuroprotective effect, NMP application 10 min before CCI was assessed in a separate set of experiments. The bwc increased by 2.4 ± 0.2% 24 h after CCI in the control group; application of NMP at a concentration of 0.5% significantly reduced edema formation (1.8 ± 0.1%, *p* = 0.01; Fig. 3A). There was a trend toward improvement in respect to weight loss (Fig. 3B, figure contains data from both the lesion size and the brain edema groups, therefore *n* = 16 for control, NMP 0.5, and 1%) and neurological function (Fig. 3C, figure contains data from both the lesion size and the brain edema groups, therefore *n* = 16 for control, NMP 0.5, and 1%). Application of NMP 1 h after CCI reduced lesion volume by trend (Fig. 4), whereas application 3 h after trauma had no significant effect. Application of NMP at concentrations up to 10% had no significant effect on CBF, MAP, ICP, or heart rate (Fig. 5A–D) or blood gas analysis (Table 1).

**Table 1.** Arterial Blood Gas Analysis

**pH**		7.17 ± 0.01
**pCO_2_**	Mm Hg	45.8 ± 23
**pO_2_**	Mm Hg	130.6 ± 2.1
**Na^+^**	Mmol/L	148.3 ± 2.5
**K^+^**	mmol/L	6.24 ± 0.3
**Ca^++^**	mmol/L	1.15 ± 0.25
**HCO_3_^-^**	mmol/L	16.5 ± 8.3
**BE**	mmol/L	-11.8 ± 6.7
**etCO_2_**	mmol/L	17.9 ± 9.1
**O_2_ Sat**	%	97.8 ± 0.2

BE, base excess.

## Discussion

Despite decades of research, therapeutic options for TBI are still very limited. Many pharmacological trials tested putatively neuroprotective compounds, and many of the substances evaluated need to be dispersed in amphiphilic solvents to achieve *in vivo* applicability; however, most studies do not specifically evaluate whether the solvents used may have an intrinsic effect and thus influence experimental outcomes. In the present study we therefore evaluated whether frequently used organic solvents influence secondary brain damage in a widely used model of experimental TBI. Application of DMSO, PEG 40, and Miglyol did not influence lesion volume in our mouse CCI model, whereas NMP significantly reduced brain edema and lesion volume.

DMSO has previously been reported to be neuroprotective in different models of experimental TBI^14–16^ in various species; it has also shown to reduce lesion size following cerebral ischemia,^17,18^ a major component of the pathophysiology of secondary post-traumatic brain damage. However, the DMSO concentrations used in these studies were exceedingly high (33–100%) and had no clinical or pre-clinical relevance. Further, the effect was not consistent throughout the studies.^19^ More recent studies focusing on behavioral and outcome measures after TBI did not find an effect of DMSO at 15%,^20^ a dose higher than necessary for solving most compounds. It is therefore not surprising that the markedly lower concentrations used in this study did not alter structural post-traumatic brain damage, making low-dose DMSO a favorable solvent for TBI studies.

PEG is another widely used vehicle in multiple pharmacological studies assessing effects on acute neuronal damage. At high molecular weight (PEG 2000 and more) it seems to be protective in models of traumatic brain injury^21–24^ and spinal cord trauma,^25–27^ putatively by resealing the disrupted neuronal membrane and/or antioxidative properties. Further, PEG's molecular weight has an important impact on its biological activity. Studies using PEG at lower molecular weight did not detect effects on post-traumatic brain damage when PEG was used as a solvent.^28,29^ At a molecular weight of 40, a concentration well suitable for dissolving most agents, PEG did not influence structural brain damage and seems therefore an appropriate choice for neuroprotection studies.

Miglyol 812 is a mixture of medium-chain triglycerides (decanoyl- and octanoyl-glycerides) that is commonly used as a solvent in the cosmetic and pharmaceutical industry. Its application as a vehicle in experimental studies has been repeatedly reported,^30–33^ and also in the context of focal cerebral ischemia.^34,35^ The effect of Miglyol on brain damage was, however, not specifically assessed as both published studies on cerebral ischemia lack a vehicle control without Miglyol. In the present study, the solvent was used for the first time in the setting of experimental TBI. As i.p. injection did not affect lesion volume, Miglyol makes a suitable solvent option for experimental TBI studies.

Like Miglyol, NMP is used for a wide range of applications in the cosmetic and pharmaceutical industry^36,37^ and is considered safe in adults.^38,39^ Its effects on brain damage after an insult has, however, not been assessed yet. So far, NMP (100%) has only been used in two studies evaluating different solvents for agents used to treat cerebral aneurysms. In these studies NMP was found to lead to less vasospasm than other substances tested and had no major side effects after intra-arterial injection in pigs.^40,41^ In our study, low-dose NMP unexpectedly reduced lesion volume 24 h after CCI, making a vehicle control without solvent recommendable for experiments to establish the solvent's intrinsic effect. NMP also significantly attenuated brain edema formation when applied before CCI without, however, having a significant effect on functional outcome 24 h after TBI. This is most probably due to the early time-point at which neurological function was assessed. It is possible that positive effects of lesion volume and brain edema reduction translate to improvement of functional outcome only at later time-points after TBI, as previously evidenced in other studies.^11,13^ NMP has been shown to have anti-inflammatory effects: in an *in vitro* setting it reduced the concentrations of tumor necrosis factor alpha (TNF-α), interleukin (IL)-1β, IL-6, and inducible nitric oxide synthase (NOS), as well as cyclo-oxygenase 2 (COX-2).^43,44^ It is speculated that this is mediated by suppressing nuclear factor kappa-light-chain-enhancer of activated B cells (NF-κB) signaling.^42^ Inflammation is a major component of acute and chronic post-traumatic brain damage.^44,45^ The mediators previously shown to be influenced by NMP have all been found to contribute to post-traumatic brain damage.^46–55^

Another possible mechanism of action is explained by the molecular structure of NMP: After TBI, the release of free radicals contributes to the evolution of secondary brain damage.^5,56,57^ Like many of the radical scavengers successfully tested in the context of (experimental) TBI,^58–60^ NMP contains an annular structure that might be essential for scavenging free radicals and therefore ameliorating secondary brain injury.

## Conclusion

DMSO, PEG 40, and Miglyol are suitable solvents for pharmacological studies in CCI TBI as they did not show an intrinsic activity that may interfere with results. NMP, another widely used and potent solvent that was previously not evaluated in the context of brain damage, was shown to have an intrinsic neuroprotective potential. This should be taken into account when considering its use in studies of TBI, for example, by adding a vehicle control without solvent. The neuroprotective potential makes NMP a promising candidate for therapeutic use in TBI. Of note, the present study assessed an early time-point after TBI that is very commonly used in experimental TBI studies. As it is becoming more and more obvious that TBI-induced brain damage evolves over much longer periods than previously thought (Mao et al.^61^), potential long-term effects of NMP will have to be evaluated. Hence, additional studies need to be performed to further characterize its effects on long-term lesion progression and its mechanisms of action.

## Abbreviations Used

ANOVA: analysis of variance
bwc: brain water content
CBF: cerebral blood flow
CCI: controlled cortical impact
COX-2: cyclo-oxygenase 2
CPP: cerebral perfusion pressure
DMSO: dimethylsulfoxide
i.p.: intraperitoneal
ICP: intracranial pressure
IL: interleukin
MAP: mean arterial pressure
Miglyol: Miglyol 812
NF-κB: nuclear factor kappa-light-chain-enhancer of activated B cells
NMP: *N*-2-methyl-pyrrolidone
NOS: nitric oxide synthase
NSS: Neurological Severity Score
PBS: phosphate buffered saline
PEG 40: polyethyleneglycol 40
SD: standard deviation
TBI: traumatic brain injury
TNF-α: tumor necrosis factor alpha
